# Supplementary material for: Progressive decomposition of infrared and visible image fusion network with joint transformer and Resnet
Source: PLoS One. 2025 Aug 22;20(8):e0330328. doi: 10.1371/journal.pone.0330328 (PMC12373181; doi:10.1371/journal.pone.0330328)
Supplement: Attachment — Submitted filename: Response to Reviewers.docx [file pone.0330328.s003.docx]

Modification Explanation

Firstly, we appreciate the comments of the reviewers very much. The comments are beneficial to improve the quality of this research paper. Then, we have made modifications with a point-by-point response to the reviewer’s comments as follows:

Reviewer #1:

1. Originality of the topic:
   • As described in Sections 1 and 3, the study investigates the integration of Transformer and ResNet techniques with progressive decomposition for infrared and visible image fusion. Various studies have discussed similar topics (Using Transformer, ResNet, and other techniques for decomposing image fusion). Thus, the topic of this study is not original in its field. It builds upon already existing studies, and its findings contribute to the field.
   • The researchers discuss the advancement of their approach (in Section 5), yet they do not clearly state how their work differs significantly from other similar studies. Clarifying the novelty of the study is important.

Thank you very much for your careful review of our paper. Thank you for your comments. Based on your suggestions, in Section 5 we highlighted the differences from other similar works to clarify the novelty of this study.

2. Non-published results:
• In the manuscript, the researchers admit that the study has not been published, and no specific funding was received.
(The use of the datasets, e.g., RoadScene, proves that the results of the study have not been published before).

Thank you very much for your careful review of our paper. Thank you for your comments. The manuscript is supported by multiple grants, and this will be specifically indicated again during the submission process.

3. Standard and detailed description of experiments, statistics, and analyses:
• The databases (e.g., RoadScene, MSRS), fusion methods, metrics (e.g., SD, VIF), settings (e.g., software and hardware), and parameters used in the study are clearly specified and systematically described.
• The study lacks a statistical significance test (e.g., p-values) to validate the superior performance of the proposed architecture over other approaches (e.g., SOTA).
Thank you very much for your careful review of our paper. Thank you for your comments. In our manuscript, the quantitative metrics include EN (Entropy) and $Q_{AB/F}$, which are commonly used evaluation measures (or P-value metrics) in the field of image fusion.

4. Appropriate conclusions supported by data:
   • The conclusions of the study are well-supported by quantitative metrics (e.g., figure 10), qualitative comparisons (e.g., figure 11), and with the experimental findings (Section 5).
   • The study’s findings have made a valuable contribution to the field.

Thank you very much for your careful review of our paper. Thank you for your comments. Thank you very much for your positive feedback on our manuscript.

5. Intelligible Standard English:
• The Language is intelligible Standard English, with precise technical terms.
• The manuscript is well-organized. Its content structure is logical: introduction, previous studies (review of IVIF methodologies and discussion of the framework), research method, experiments, and conclusion.
• Minor grammatical refinements are needed to clarify the meaning (e.g., "visual fusion images" could be “fused visual images”).

Thank you very much for your careful review of our paper. Thank you for your comments. As requested, certain grammatical issues in the manuscript have been revised.

6. Standards for the ethics of experimentation and research integrity:
• In the manuscript, the authors admit that there are no human or animal participants in the study (using N\A).
Thank you very much for your careful review of our paper. Thank you for your comments.

7. Reporting guidelines and community standards for data availability.
• Minor issue with data availability: in the manuscript, the researchers state that “all data are fully available without restriction” and “if the manuscript is lucky enough to be accepted, the URL of the data and code will be made public”.

Thank you very much for your careful review of our paper. Thank you for your comments. All data and code from the manuscript will be made public and are fully accessible!

Reviewer #2:

1. Problem: The description of the three-stage method is too general and does not explain the technical connections between each stage (such as how LT decomposes coarse features into basic and detailed features). Suggestion: (1) Use formulas to simplify the key steps, such as "coarse feature decomposition: \ (F2 {coarse}=CNN (I2 {ir}, I2 {vi}) \), \ (F2 {base}, F2 {detail}=LT (F2 {coarse}) \)". (2) Supplement the data flow between modules, for example, in Stage II, the features output by the ResB+LT module are fused by SIFM and input into INN to extract details.

Thank you very much for your careful review of our paper. Thank you for your comments. (1) As per your suggestion, I have subdivided and elaborated on the formula section in Section 3. Additionally, the image features processed by the backbone network are presented in Table 2. Please refer to the modifications marked in blue. (2) Based on your suggestion, I have supplemented the data flow between modules in Section 2. Please refer to the blue-marked section 2.4.

2. Problem: The defect analysis of comparative methods such as CCDFuse and PSFusion remains at the level of phenomenon description, lacking theoretical attribution (such as the mathematical explanation of "simple addition strategy leading to feature conflicts"). Suggestion: (1) The existing methods for quantifying feature distribution distance are insufficient, such as "The KL divergence of CCDFuse features is 0.82, significantly higher than the 0.45 of our method". (2) Based on the CKA results in Figure 1 (c), it can be concluded that "traditional symmetric fusion leads to a 42% misalignment of IR and VI feature spaces".

Thank you very much for your careful review of our paper. Thank you for your comments. Following your suggestions, we have included Table 1 to present the computed average Kullback-Leibler (KL) divergence and Centered Kernel Alignment (CKA) values between the fused and original images, obtained through Python-based calculations. A comprehensive interpretation of these quantitative results is provided in the blue-highlighted explanatory notes preceding the table.

3. Problem: The motivation part does not establish a causal relationship between "feature space differences" and "asymmetric fusion", lacking a logical chain. Suggestion: (1) Additional derivation: "As VI achieves deep semantic features at 12 layers (Figure 1 (a)), while IR requires 32 layers (Figure 1 (b)), design a cross layer fusion strategy VI-Encor-1 → IR-Encor-2". (2) Introduce transfer learning theory and explain that 'VI features serve as a teacher model to accelerate the distillation of IR features through SE blocks'.

Thank you very much for your careful review of our paper. Thank you for your comments. We appreciate your feedback and have accordingly enhanced the description of our network architecture on Page 4. This includes detailed explanations of the encoder-decoder processing pipeline for both infrared and visible light images. Specific technical details are highlighted in blue for easy reference. Additionally, the following section will present a comprehensive flowchart to visually demonstrate the network's data flow and feature transmission mechanisms between these two modalities.

4. Problem: Lack of network architecture details. (1) The structural difference between IR UNet and VI UNet only mentions the "SE block", without specifying its specific position in the encoder (such as insertion after ResB1). (1) The mathematical expression for asymmetric fusion is missing, such as' the fusion function of VI-Encorder-i and IR-Encoder - (i+1) is undefined '. Suggestion: (1) Use a table to compare the layer structures of IR UNet and VI UNet, and label the SE block positions (such as "Insert one SE block after ResB1~ResB4"). (2) Define the fusion function: \ (F2 {fuse}=\ text {Attention} (F2 {vi} ^ i) \ otimes F2 {ir} ^ {i+1}+F2 {ir} ^ {i+1} \), where \ (\ text {Attention} \) is the channel attention mechanism.

Thank you very much for your careful review of our paper. Thank you for your comments. (1) Following your suggestions, I have reformulated Equations (1)-(6) where A denotes the visible light image and B represents the infrared image. These equations explicitly describe the hierarchical encoding process between the two modalities. (2) Please refer to the added Table 2. The feature information structures extracted at each level of the backbone network for infrared and visible light images are the same, only the content differs. The two original images are simultaneously fed into the backbone network, and we will provide the specific code. The fusion function at each level—for example, the base layer employs the SIFM module for fusion—follows the detailed fusion rules described in reference [11]. The detail layer fusion adopts the SDFM module, with specific details referenced in Equations (10) and (11). Additionally, the integrated feature information is fed into the D2FM module, with specific processing details referenced in Equations (12)-(16). The Unet fusion is based on Equations (17)-(22).

5. Problem: The freezing range of parameters for two-stage training is unclear, such as whether to freeze decoder parameters in Stage II. Suggestion: (1) Use a flowchart to illustrate the training sequence and label "Stage I trains VI UNet first (freezes LT module), Stage II trains IR UNet (freezes VI UNet encoder)". (2) Clarify the scope of parameter updates: "When training the fusion model, only the parameters of DF2M and SIM are updated, and the parameters of other modules are frozen.

Thank you very much for your careful review of our paper. Thank you for your comments. Per your request, we have supplemented the manuscript with Fig. 6 and 7, which provide a comprehensive illustration of the infrared (IR) and visible light (VIS) image encoding/decoding architecture. Detailed technical specifications are annotated in blue text beneath Fig. 7. During network training, our loss function simultaneously constrains: (1) intermediate base and detail features across network layers, and (2) the final fused image output. This multi-objective optimization framework enables coordinated parameter adjustment across all modules depicted in Figures 6 and 7. For implementation specifics, please refer to the accompanying Python code where these processes are concretely implemented. The loss function is not displayed in the framework diagram, please refer to Equations (28)-(32).

6. Problem: Insufficient depth of ablation experiment. (1) The attention weight distribution of SE blocks has not been analyzed (such as the proportion of channel attention for different modal features). (2) Lack of comparison using only cross scale fusion (without guidance mechanism) makes it impossible to separate the contributions of innovative points.

Suggestion: (1) Visualize the attention map of the SE block, such as "After VI guidance, the temperature related channel weights in the channel attention of IR features increase by 27%". (2) Add the ablation group "- w/o Guidance" and compare its MS-SSIM difference with the complete model (as shown below, decrease by 0.041).

Thank you very much for your careful review of our paper. Thank you for your comments. We sincerely appreciate your valuable suggestions. Our framework follows an architecture similar to the 2023 paper we cited (L. Tang et al, Rethinking the necessity of image fusion in high-level vision tasks: A practical infrared and visible image fusion network based on progressive semantic injection and scene fidelity, Information Fusion, 99 (2023) 101870.) : rather than using phased training, we process infrared and visible images in parallel through the backbone network for progressive inter-layer feature extraction, storing all intermediate features in GPU memory before performing subsequent integration of both base and detail information through a U-Net-inspired decoder that enables bottom-up aggregation to produce the final fused image. For ablation studies, we conducted comparative analyses of three core components—the detail feature extraction module, the backbone's LT module, and the INN-based detail processor—with additional quantitative comparisons provided in Table 4.

7. Problem: The contradictions in the indicators have not been explained. In Table I, the PSNR (13.106) of MMA UNet in MSRS is lower than that in DDFM (13.485), but the SSIM is higher, and the reason is not explained. Suggestion: (1) Analysis: "DDFM preserves more noise, resulting in artificially high PSNR, while our method achieves more natural detail reconstruction through structural constraints (SSIM=0.582). (2) Supplementary visual comparison: In Figure 3, mark the noise area of DDFM with a red box and compare it with the detail preservation effect of the method proposed in this paper.

Thank you very much for your careful review of our paper. Thank you for your comments. I am very sorry and also quite confused. In my submitted manuscript, Table 1 presents the results of semantic segmentation testing on the MFNet dataset, not PSNR and SSIM metrics on the MSRS dataset. Additionally, our manuscript does not mention the DDFM method, or perhaps I misunderstood your point? Could you please clarify in more detail? Thank you very much!

8. Problem: Key terms are not standardized. (1) The definition of "common features" is vague and the CKA similarity threshold is not specified (such as "features with CKA>0.6 are common features"). (2) The concepts of "asymmetric fusion" and "cross scale fusion" are confused and not clearly distinguished. Suggestion: (1) Define in the terminology table: "Common features: features with cross modal CKA similarity>0.6; Private features: Features with CKA<0.3. (2) Clear conceptual boundaries: "Asymmetric refers to the unequal hierarchy of fusion layers (such as VI-1 → IR-2), while cross scale refers to multi-scale feature fusion.

Thank you very much for your careful review of our paper. Thank you for your comments. In the manuscript, I have already provided explanations for basic features and detailed features above Table 1. Additionally, the concepts of "asymmetric fusion" and "cross-scale fusion" have been revised accordingly in the text.

9. Problem: The formula symbols are not unified. In formula (1), \ (Coarse_S (A) \) is not defined, and in formula (3), \ (ResB ^ i \) is not specified as the i-th block of ResNet. Suggestion: (1) Add a comment next to the formula: "Coarse-A (\ cdot) \" represents the coarse feature extraction CNN for infrared images.(2) Unified symbol: "\ (ResB ^ i \) represents the i-th residual block of ResNet34 (i=1-4)".

Thank you very much for your careful review of our paper. Thank you for your comments. We have provided a detailed explanation of Equation (1); please refer to the updated Equations (1)-(3). The ResNet34 residual blocks in Equation (3) of the initially submitted manuscript have also been explained - see Equations (5)-(6) for details.

10. Problem: The chart information is incomplete. (1) Figure 1 does not indicate the meaning of the coordinate axis (e.g. the x/y axis represents the number of network layers, and the color represents CKA similarity). (2) Figure 3 shows the data flow between unmarked modules (such as the input-output feature dimension of SIFM). Suggestion: (1) Supplementary note to Figure 1: "The warm colored region in (c) represents the high similarity layer (CKA>0.5) between IR and VI features. (2) Add arrow annotation in Figure 3: "ResB+LT → SIFM → INN" feature transfer path.

Thank you very much for your careful review of our paper. Thank you for your comments. (1)We have calculated the average CKA values for the results obtained by the three methods below Figure 1. Due to the relatively low CKA values between the fused images and infrared (IR) images, the average CKA appears not very high. In Table 1, we provide a detailed explanation of the CKA similarity—please refer to the blue highlighted section for further clarification. (2) The input and output dimensions of the backbone network's features are presented in the newly added Table 2. In the newly added Figure 6, we illustrate the detailed feature transfer path. For example, the infrared image passes through ResB1+LT in the backbone network to obtain $F_{A}^{3}$ ([4, 64, 64, 64]), which is then processed by INN (leveraging its powerful detail extraction capability) to generate $F_{detail,A}^{3}$—a feature representation of the same dimensions.

11. Problem: The tuning method for \ (\ alpha=10 \) and \ (\ beta=0.5 \) in formula (5) is not specified, and the value basis for \ (\ varepsilon \) in formula (31) is missing. Suggestion: (1) Add: "\ (\ alpha, \ beta \) Use grid search to select the optimal value from {0.1, 1, 10}. (2) Explanation: "\ (\ varepsilon=1e-4 \) is used to prevent the denominator from being zero, refer to the setting in [36].

Thank you very much for your careful review of our paper. Thank you for your comments. (1) After carefully reviewing the initially submitted manuscript, I was quite perplexed. Formula (5) demonstrates the process of integrating fundamental information, yet it does not involve parameters alpha or beta. Moreover, through meticulous examination of all equations, I confirmed that parameter beta does not appear anywhere in our framework. All parameters in our system have been carefully optimized through extensive experimental validation. (2) Based on your suggestion, I have provided clarification regarding ε (varepsilon) in Equation (31).

12. Problem: Limitations of dataset and comparison methods. (1) Untested TNO dataset (including more complex lighting scenarios), unable to verify robustness under extreme conditions. (2) The comparison method lacks the latest algorithm from 2024 and lacks timeliness. Suggest to supplement relevant experiments (with a large workload, selectively solve according to the actual situation)

Thank you very much for your careful review of our paper. Thank you for your comments. (1) The infrared and visible image pairs selected in Figure 1 and 16 are those with complex external environments chosen from the TNO dataset. (2) As per your request, we have added a 2025-published paper on a deep learning-based fusion framework, referred to as the LFDT fusion method. We removed DenseFuse from the comparative methods and incorporated the LFDT fusion approach instead. We have reorganized the experimental section in Section 4. Please refer to Figures 8-15 and Table 3 for details. All qualitative and quantitative comparisons have been updated with the assistance of Python code and Matlab tools. Since the figures and tables could not be annotated, we have left them unmarked. Kindly review them at your convenience. Thank you very much!
